# Bacterial community profiling and identification of bacteria with lignin-degrading potential in different gut segments of African palm weevil larvae (*Rhynchophorus phoenicis*)

**DOI:** 10.3389/fmicb.2024.1401965

**Published:** 2025-01-03

**Authors:** Jessica Lenka, Enrique González-Tortuero, Shweta Kuba, Natalie Ferry

**Affiliations:** ^1^School of Science, Engineering and Environment, University of Salford, Salford, United Kingdom; ^2^School of Life Sciences, Faculty of Natural Sciences, Keele University, Staffordshire, United Kingdom; ^3^Department of Applied Sciences, Faculty of Health and Life Sciences, Northumbria University, Newcastle upon Tyne, United Kingdom; ^4^School of Health and Life Sciences, Teesside University, Middlesborough, United Kingdom

**Keywords:** African palm weevil, lignin-degrading, 16S rRNA, bacterial community, lignocellulose, foregut, midgut, hindgut

## Abstract

The microbiota within the guts of insects plays beneficial roles for their hosts, such as facilitating digestion and extracting energy from their diet. The African palm weevil (APW) lives within and feeds on the high lignin-containing trunk of palm trees; therefore, their guts could harbour a large community of lignin-degrading microbes. In this study, we aimed to explore the bacterial community within the gut of the APW larvae, specifically with respect to the potential for lignin degradation in various gut segments as a first step to determining the viability of mining bacterial lignin-degrading enzymes for the bioconversion of lignocellulosic biomass to biofuels and biomaterials. Bacterial metagenomic DNA was extracted from the foregut, midgut, and hindgut of larvae of the APW, and the V3–V4 hypervariable region of the 16S rRNA gene was sequenced using the Illumina MiSeq platform. The generated data were analysed and taxonomically classified to identify the different bacterial phylotypes within the gut community cumulatively and per gut segment. We then determined the presence, diversity, and abundance of bacteria associated with lignin degradation within each larval gut compartment as a basis for suggesting the gut segment(s) where lignin degradation occurs the most. All sequences were classified and belonged to the bacterial kingdom. *Firmicutes* (54.3%) and *Proteobacteria* (42.5%) were the most dominant phyla within the gut, followed distantly by *Bacteroidota* (1.7%) and *Actinobacteriota* (1.4%). *Enterococcus*, *Levilactobacillus*, *Lactococcus*, *Shimwellia*, *Megasphaera*, *Klebsiella*, *Pectinatus*, *Salmonella*, *Lelliotia*, and *Enterobacter* constituted the most abundant genera found across all gut segments. The foregut and midgut had many similar genera, whilst the hindgut appeared unique. Overall, 29.5% of total gut bacteria comprising 21 genera were lignin degraders found predominantly in the *Firmicutes* and Proteobacteria phyla (56.8 and 39.5%, respectively), then moderately in *Actinobacteriota* (2.5%) and *Bacteroidota* (1.1%). The most abundant ligninolytic genera were *Levilactobacillus* (46.4%), *Klebsiella* (22.9%), *Enterobacter* (10.7%), *Lactiplantibacillus* (5.9%), *Citrobacter* (2.2%), *Corynebacterium* (1.8%), *Paucilactobacillus* (1.8%), *Serratia* (1.5%), *Bacteroides* (1.1%), and *Leucobacter* (1.0%) found in different amounts in different gut compartments. The foregut had the most diverse and highest abundance of lignin-degrading phylotypes, and we present reasons that point to the foregut as the main location for the depolymerization of lignin in the APW larval gut.

## 1 Introduction

Beneficial associations between insects and their gut microbial inhabitants, especially concerning the host’s nutrition, can be exploited for biotechnological applications (Harrison et al., 2021; Rajagopal, 2009; Chukwuma et al., 2021). Wood-feeding insects are known to be able to digest and utilise plant biomass by the synergistic association they enjoy with the microorganisms that inhabit their gut (Ali et al., 2019; Chew et al., 2018; Scully et al., 2013; Chauhan, 2020; Kougias et al., 2018). Recently, much attention has been given to understanding the composition of the inhabitant microbes and how they are naturally adapted to facilitate these bioconversion processes (Prasad et al., 2018; Ransom-Jones et al., 2017).

Molecular techniques such as PCR and high-throughput sequencing have facilitated the studies of microbial communities without depending on the ability to culture individual members of the community, as the optimum conditions for growing different species of microbes vary or are yet undetermined for most species (Lazarevic et al., 2016; Stewart, 2012). Structural survey methods of studying microbiomes aim to identify the taxonomic profiles of the study environments concerning the types of microorganisms present (diversity) and their amounts (abundance or richness), from which functional capability can be predicted if desired (Kunath et al., 2017; Knight et al., 2018).

There is a plethora of studies that have investigated insect gut bacterial compositions using the 16S rRNA amplicon sequencing technique and have primarily identified *Proteobacteria*, *Firmicutes*, *Actinobacteria*, and *Bacteroidota* as the predominant bacterial phyla in insect guts, amongst many other species and environment-specific findings (Prasad et al., 2018; Scully et al., 2013; Do et al., 2014; Ali et al., 2019; Bozorov et al., 2019). The V3–V4 hypervariable region of the 16S rRNA gene has been targeted in many published sequencing studies of phylogenetic and taxonomic classification of insect gut microbiomes (Ben Guerrero et al., 2016; Lazarevic et al., 2016; García-López et al., 2020; Lluch et al., 2015; Chew et al., 2018). Some of these studies have also pointed out the fact that the gut microbiome of insects is non-static and influenced by factors such as environment (Yun et al., 2014), host phylogeny (Franzini et al., 2016; Mohammed et al., 2018), developmental stage and season (Valzano et al., 2012; Jia et al., 2013), nutrition and diet (Montagna et al., 2015; Muhammad et al., 2017; Ben Guerrero et al., 2016), gut physiology and conditions for pH, temperature, and oxygen availability (Egert et al., 2003; Chew et al., 2018). Regardless, there are core members of the community that are only mildly influenced by such factors that may persist, thereby defining the most fundamental functions performed by the microbiome (Pal and Karmakar, 2018; Reich et al., 2018; Franzini et al., 2016; Ben Guerrero et al., 2016).

Research on bacteria capable of lignin degradation has only recently gained much attention, as most studies of microbial lignin degradation have centred on fungi (Bugg et al., 2011a). The ability to directly degrade and modify lignin has been shown in several bacterial phyla such as *Proteobacteria*, some *Firmicutes*, and *Actinobacteria*, the majority of which were obtained from the guts of ruminants, termites, and other wood-feeding insects (Bugg et al., 2011b; Huang et al., 2012; Arumugam et al., 2014; Bugg and Rahmanpour, 2015; Kassim et al., 2016; Janusz et al., 2017). Other bacteria identified and shown to possess lignin-degrading or modifying abilities from several other research outcomes have been compiled and are presented in Table 1.

**Table 1:** Lignin-degrading bacterial genera identified from literature reports.

Phyla	Genus	Species/strain	References
Protobacteria			
	*Pseudomonas*	*Strain Q18*	Yang et al. (2018)
		*Fluorescens*	Rahmanpour and Bugg (2015)
		*Putida*	Xu et al. (2018)
	*Burkholderia*	sp. *H1*	Yang et al. (2018)
		*Strain ISTR5 (R5)*	Morya et al. (2019)
	*Klebsiella*	*Pneumoniae*	Xu et al. (2018), Gaur et al. (2018), and Yadav and Chandra (2015)
		*Aerogenes TL3*	Tu et al. (2024)
	*Xanthomonas*	*NA*	Jiménez et al. (2016)
	*Ochrobactrum*	*Tritici*	Xu et al. (2018)
		sp.	Rashid and Bugg (2021)
	*Acinetobacter*	*Johnsonii LN2; Iwoffi LN4*	Xiong et al. (2020)
	*Enterobacter*	*Lignolyticus SCF1*	DeAngelis et al. (2013)
		*Cancerogenus*	Özer et al. (2019)
		*Ludwigii*	Chen et al. (2018)
	*Citrobacter*	*Sedlakii, farmeri*	Özer et al. (2019)
		*Freundii (FJ581026) and* sp. *(FJ581023)*	Chandra and Bharagava (2013)
	*Serratia*	sp. *JHT01, liquefaciens PT01*	Tian et al. (2016)
		*Quinivorans AORB19*	Ali et al. (2024)
		sp. *AXJ-M*	An et al. (2023)
		*Proteamaculans*	Ali et al. (2022)
	*Escherichia*	*O157 Asp143*	Liu et al. (2017)
	*Sphingobium*	*Lignivorans* sp. *nov.*	Allemann et al. (2023)
	*Sphingomonas*	*Paucimobilis SYK-6*	Masai et al. (2007)
	*Comamonas*	sp. *B-9*	Chen et al. (2012)
		*Serinivorans*	Sethupathy et al. (2023)
		*Testosteroni FJ17*	Wang et al. (2022)
	*Pantoea*	*Ananatis*	Shi et al. (2015)
	*Pandorea*	sp. *ISTKB*	Kumar et al. (2017b)
	*Delftia*	sp. *JD2*	Morel et al. (2016)
	*Cupriavidus*	*Basilensis*	Shi et al. (2013)
Actinobacteria			
	*Streptomyces*	*Viridosporus*	Ramachandra et al. (1988) and Crawford et al. (1983)
		sp. *S6*	Riyadi et al. (2020)
	*Rhodococcus*	*Jostii RHA1*	Ahmad et al. (2011)
		*Pyridinivorans CCZU-B16*	Chong et al. (2018)
	*Azotobacter*	sp. *HM12*	Morii et al. (1995)
	*Nocardia,*	sp. *DSM 1069*	Eggeling and Sahm (1980)
		*Albiluteola* sp. nov.	Shan et al. (2022)
	*Corynebacterium*	*Glutamicum*	Merkens et al. (2005)
	*Mycobacterium*	sp. *strain CG-2*	Häggblom et al. (1988)
	*Saccharomonospora*	*Viridis DSM 43017*	Yu et al. (2014)
	*Thermomonospora*	*Mesophila*	McCarthy et al. (1986) and Godden et al. (1992)
	*Microbacterium*	sp.	Taylor et al. (2012) and Tian et al. (2014)
	*Leucobacter*		Chauhan (2020)
Firmicutes			
	*Bacillus*	*Aryabhattai BY5*	Xiong et al. (2020)
		*Subtilis*	Yadav and Chandra (2015)
		*Ligniniphilus L1*	Zhu et al. (2017)
Bacteroidota			
	*Bacteroides*		Taylor et al. (2012)
	*Vogesella*	sp.	Woo et al. (2017)

A list of different lignin-associated bacterial genera (grouped according to phyla) identified through a literature search of published articles.

Despite the increase in research on gut microbial communities, studies about how these communities are organised within each gut compartment using culture-independent methods are not readily available, as most gut bacterial diversity studies have been about the whole gut communities or are taxa-specific. This presents a need for broader and systematic identification of the diversity in each segment of the gut of these insects to provide a wider description of the microbial community and relate the contribution of members of the community in each gut segment to the overall host’s metabolism, adaptability, and survival (Engel and Moran, 2013; Poelchau et al., 2016).

Industrial-scale bioprocessing of lignocellulosic biomass as viable substitutes to fossil-based sources is plagued by a lack of efficient pre-treatment and lignin valorization strategies that align with the global outcry for green and sustainable processes to minimise environmental damage and their climate change consequences. In biorefineries, substituting currently used chemical and thermophysical methods of biomass pre-treatment with biological enzyme-based methods will go a long way in alleviating costs and slowing down climate change. Given this, researchers have prioritised exploring natural biomass-utilising systems such as the guts of wood-feeding insects to maximise the chances of isolating the most efficient candidate enzymes of microbial origin, which serve to facilitate the breakdown of the host’s lignocellulose-rich diet for potential application in industrial bioconversion of lignocellulose to biobased products as alternatives to chemical methods (Olsson, 2016; Brown and Chang, 2014). Detailed studies surrounding the enzymology of ligninolytic enzymes are being intensified, and several classes of enzymes potentially possessing ligninolytic activity have been identified from lignin-degrading fungi and bacteria (Fisher and Fong, 2014).

The African palm weevil (*Rhynchophorus phoenicis*) belongs to the Curculionidae family of beetles (Coleoptera). It is an important pest affecting mostly oil palm trees in Nigeria, Cameroon, and other subtropical African countries where it is found. Other host plants of this insect include sugar cane, coconut, raffia palm, and the sago palm (Omotoso and Adedire, 2007; Mba et al., 2017). The weevil lives their entire life cycle within the trunk of palm trees, feeding on the palm tissue, which has been reported to have high lignin content (Al-Zuhair et al., 2015; Fadele et al., 2017; Ameh et al., 2016; Nasser et al., 2016; Bensah et al., 2015), and the larval stage of development is the most destructive stage of this insect (Bamidele et al., 2013; Harris et al., 2015; Angzzas et al., 2016; Omotoso, 2013). Despite this lignin content, the APW overcomes the lignin barrier as it excavates and burrows deeply into the interior of the trunk of healthy trees, leading to their eventual destruction. In such a sense, the weevils most likely benefit from the synergistic relationship with their gut microbiota that enables them to degrade lignin (Geib et al., 2008), hence our interest in profiling the bacteria inhabiting the gut of the insect with respect to the potential for lignin degradation. Thus, these insect guts could be reservoirs for novel lignocellulose/lignin-degrading enzymes that could be explored for increased efficiency of industrial plant biomass bioconversion processes into energy and material products. To the best of our knowledge, there has not been any comprehensive investigation or exploration of the gut microbiota of *Rhynchophorus phoenicis*. Therefore, in this study, we present the first attempt at exploring the bacterial community within the gut of the APW larvae, specifically with respect to the potential for lignin degradation as a first step to determining the viability of mining bacterial lignin-degrading enzymes.

## 2 Methods

### 2.1 Field collection of APW larvae

**Figure 1:**
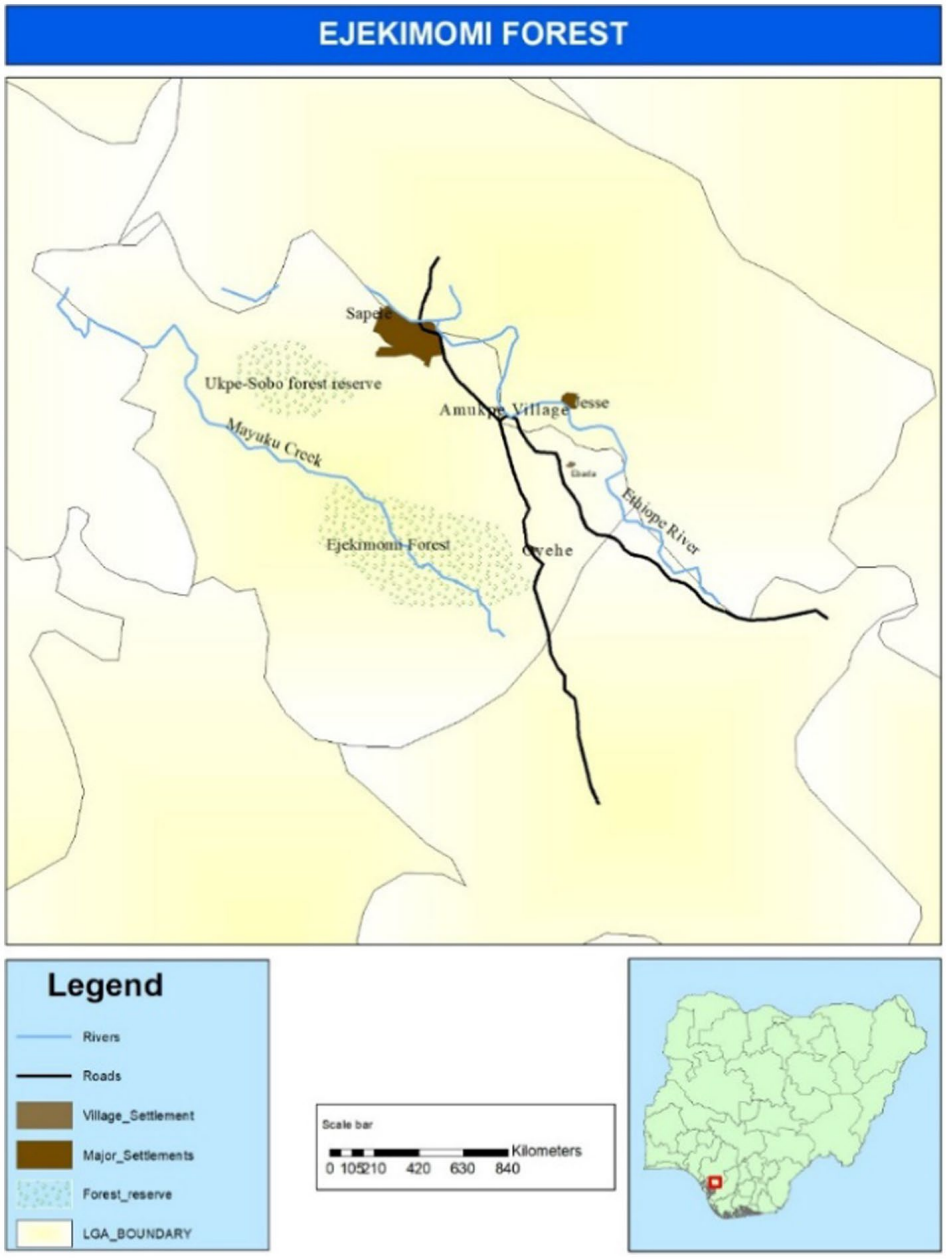
Map showing the location of Ejekimomi forest reserve area in Amukpe village of Sapele town, in Delta state Nigeria where African palm weevil larvae were collected.

Actively feeding larvae of the African palm weevil (*R. phoenicis*) were collected from different freshly felled palm tree trunks at the Ejekimomi forest reserves of Amukpe village in Sapele, Delta state, Nigeria (5°52′29.9″N 5°42′14.3″E) in 2019 (Figure 1).

Figure 1 is a map of the Ejekimomi forest reserve area in Amukpe village, Sapele town, in Delta State, Nigeria, where African palm weevil larvae were collected. *R. phoenicis* larvae were identified based on their morphological characteristics. The larvae were washed in sterile water to remove dirt and chopped tree particles; surface sterilisation was conducted using 70% ethanol and 10% bleach, and later, it was rinsed a second time in distilled water (Hammer et al., 2015; Mohammed et al., 2018). Larvae were packaged in sterile containers in the laboratory-prepared and sterilised NAP buffer (Camacho-Sanchez et al., 2013). The samples were stored at 4°C until dissection and DNA extraction (see Supplementary Data 2.0).

#### 2.1.1 Ethics statement

Ethical clearance is unnecessary for work on insects (Franzini et al., 2016). Also, *Rhynchophorus phoenicis* has not been listed as protected or endangered in national or regional laws. However, ethical approval was obtained to undertake this research due to the Nagoya protocol which emphasises the need for agreement and benefit sharing when accessing genetic materials from a different country as enshrined in the provisions of the biodiversity convention (Ajai, 1997; Omotoso, 2013). The sample collection was done in open and unprotected forests with the agreement and support of the local community.

### 2.2 Dissection and bacterial DNA extraction from larval guts of APW

Stored larvae were removed from the NAP buffer and dried in a Petri dish. Ten randomly selected larvae were cut open from the mouth to the end of the abdomen using a sterile scalpel and forceps, and the whole guts were aseptically removed separately. Each whole gut was further sectioned into “Foregut,” “Midgut,” and “Hindgut” based on the description of the boundaries of each gut segment (Omotoso, 2013). Gut tissue pieces from the same gut segments were collected in one tube each. The tissues in each tube were cut into smaller pieces, homogenised, centrifuged, and collected supernatant. The supernatant collected was split into two, making two technical replicates per gut segment sample, and bacterial DNA was extracted from these using the QIAamp DNA microbiome kit from Qiagen, UK (Cat. No. 51704), according to the manufacturer’s instructions. A negative extraction control, which contained no DNA, was prepared and processed alongside the gut segment samples. Twenty-five microliters (25 μL) of bacterial DNA were eluted from each QIAamp mini column into 1.5 mL Eppendorf tubes. The concentration and purity of the eluted DNA samples were measured using a Nanodrop spectrophotometer (Thermo Fisher, United Kingdom).

### 2.3 Library preparation and sequencing

DNA samples from the different gut segments (foregut, midgut, and hindgut samples), extraction control (control sample), and ZymoBIOMICS microbial community standard (ZYMO research, United States), which contains a well-defined bacterial composition ideal for the validation of microbiomic workflows (standard sample), were sent to Macrogen, Inc. (NGS), Seoul, Republic of Korea, for library preparation and sequencing. The V3–V4 region of the 16S rRNA gene was amplified using the primers 337F/805R (F337: 5′-GACTCCTACGGGAGGCWGCAG-3′ and 805R: 5′-GACTACCAGGGTATCTAATCC-3′). Sequencing libraries were prepared using the Herculase II Fusion DNA Polymerase Nextera XT Index Kit V2 (Illumina) according to the protocols in the Illumina 16S metagenomic sequencing library preparation guide (Part #15044223 Rev. B). The libraries were purified, quality validated, diluted to 6 nm, and pooled. Paired-end sequencing (2 × 300 cycles) was carried out on an Illumina MiSeq device (Illumina Inc., San Diego, CA, United States) according to the manufacturer’s specifications.

### 2.4 Data processing and analysis

The data file containing forward and reverse reads for each sample was imported into R-studio software version 4.1.0 (R core team 2020) and was processed following guides from the DADA2 pipeline tutorial 1.16 (Callahan et al., 2016) with slight modifications to suit our reads and desired outcome. Data pre-processing involved quality profiling, trimming, and filtering raw data to eliminate read duplicates, low-quality reads, adapters, and barcode sequences. Paired reads that passed quality processing were denoised and merged, and amplicon sequence variants (ASVs) with corresponding frequencies for each sample were generated (Callahan et al., 2016). Taxonomy was assigned to each ASV using the AssignTaxonomy function to genus level using the Silva_nr99_v138 training set database (Glöckner et al., 2017) for bacterial 16S rRNA as the reference because taxonomic assignments at species level do not yield satisfactory resolution with amplicon sequencing in most cases (Callahan et al., 2016).

The taxonomy assigned ASVs were processed using the R package *phyloseq* version 1.36.0 (McMurdie and Holmes, 2013). Each sample was identified as a “True” sample (standard, foregut, midgut, and hindgut) or a “Negative” sample (control sample). To ensure no contamination by external sources was present, the automated prevalence-based strategy in the R package *decontam* version 1.12.0 (Davis et al., 2018) was used with the phyloseq object. ASVs corresponding to sequences identified as mitochondria and chloroplast sequences were removed, and all ASVs identified to belong to the same genus were merged. The different ASVs per gut segment were visualised in KronaTools version 2.8.1 (Ondov et al., 2011).

To assess the accuracy of the sequencing and taxonomic identification procedure, a separate phyloseq object was created containing only the mock bacterial community sample. This was analysed by examining ASV counts and comparing their observed relative proportions to the expected theoretical proportions of species declared in the ZymoBIOMICS microbial community DNA standard product literature (ZymoBIOMICS^™^ Microbial Community DNA Standard instruction manual, ver1.1.5). This information was represented as a bar chart using Microsoft Excel. A comparison between the observed and expected taxa was made using a chi-square test in R version 4.4.1.

Bacterial genera with <10 ASV counts of cumulative abundances (total abundance from all gut segments) were filtered out, and only those with >10 counts were used for further analysis. The most abundant bacterial phyla and genera identified in the APW gut were presented on a pie chart plotted in Microsoft Excel. Using Microsoft PowerPoint, a Venn diagram was created to show taxa shared between the different gut segments.

To evaluate potential differences in the microbial composition amongst the different gut segments, a non-metric multidimensional scaling (NMDS) plot using Bray–Curtis dissimilarity was constructed considering the number of counts. Then, permutational multivariate analyses of variance (PERMANOVA; Anderson, 2001) were performed to assess differences in the microbial composition amongst the gut segments. The maximum number of iterations was set to 1,000 in all analyses. Additionally, a Kruskal–Wallis test was performed to evaluate potential differences in the microbial diversity amongst the different gut segments when considering the Shannon and inverse Simpson indexes. Shannon index was calculated using the natural logarithm. In case there were significant differences between gut segments, to see which pairs of segments showed significant differences, Holm–Bonferroni-corrected Dunn tests were performed. All these statistical approaches were carried out at an alpha level of 0.05 and were performed in R version 4.4.1 using the *vegan* version 2.6-6.1 (Oksanen et al., 2024), *FSA* version 0.9.5 (Ogle et al., 2023), *ggplot2* version 3.5.1 (Wickham, 2016), and *gridExtra* version 2.3 (Auguie, 2017).

All lignin-degrading bacterial genera identified within our samples were selected based on the current knowledge (Table 1), and their relative abundances by genus and gut segment were plotted using stacked column charts.

## 3 Results

### 3.1 Summary of raw amplicon sequence data statistics

A summary of the raw data generated following the sequencing of the 16S libraries on a 2 × 300 bp Illumina platform indicated a successful run with each sample having a high total number of paired end reads (except for the negative extraction control) and an average GC content of 54%. Additionally, 91% of the total reads sequenced had Phred quality scores higher than 20, whilst 82% had Phred quality scores of 30, suggesting that our dataset is very good quality data (Andrews, 2010; Table 2).

**Table 2:** Summary of the 16S rRNA sequencing data statistics.

S/No.	Sample ID	Total reads bases (bp)	Total reads	GC (%)	AT (%)	Q20 (%)	Q30 (%)
1	Control	11,468,100	38,100	54.235	45.77	91.722	83.379
2	Standard	112,710,052	374,452	53.888	46.11	91.686	83.206
3	Foregut1	165,148,466	548,666	54.300	45.70	91.372	82.707
4	Foregut2	156,689,764	520,564	54.982	45.02	91.244	82.423
5	Midgut1	145,899,516	484,716	54.812	45.19	91.098	82.325
6	Midgut2	136,404,772	453,172	54.341	45.66	91.190	82.516
7	Hindgut1	119,979,202	398,602	54.719	45.28	91.797	83.499
8	Hindgut2	119,740,208	397,808	54.729	45.27	91.430	82.780

### 3.2 Analysis of negative control sample (decontamination)

The duplicate negative control samples “Control” underwent all amplification, library preparation, sequencing, and bioinformatic analysis steps as the gut samples and were analysed for external contaminants using the R package *decontam* (Lazarevic et al., 2016). The output returned a “False” result with respect to the assumption that contaminating taxa are more likely to be present in the negative “control” sample compared to true samples. This observation, therefore, means that the “contaminant” taxa identified in the control samples are more present in the true samples than in the control. The negative control contained 42 ASVs, which were all present in the true samples and had a total abundance corresponding to just approximately 1.7% of the total taxa abundance found in the true samples. Only *Enterococcus*, *Lactococcus*, *Acinetobacter*, and *Bacteroides* were present at >0.1% each. All the other bacteria each had much lower values (<0.1%). Notwithstanding, these taxa were not removed from the true samples as contaminants as they are expected in the true samples and their abundances in the control sample are far lower than what was observed for each of these taxa in the true samples.

### 3.3 Analysis of mock microbial community standard

**Figure 2:**
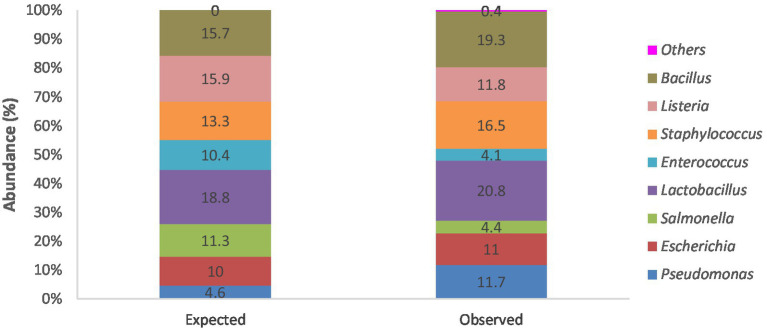
Stacked bar chart showing percentage abundances of bacterial strains expected within the mock microbial DNA community standard from theoretical data and actual observed bacterial genera identified following sequencing and analysis of the positive control “standard” sample.

The mock microbial community DNA standard made up of eight bacterial strains with various theoretical compositions for each strain was sequenced and analysed alongside the other larval gut and negative extraction control samples. This sample served as a positive control for ascertaining the fidelity of the 16S rRNA amplicon sequencing process and the performance of the data analysis pipeline used. No significant differences existed between the bacterial composition in the “observed” standard sample and the “expected” theoretical values (chi-square test: *p* = 0.2303; Figure 2).

### 3.4 Taxonomic profile of APW larval gut bacteria

#### 3.4.1 Total bacterial diversity in the APW larval gut showing percentage abundances by phyla and genera

**Figure 3:**
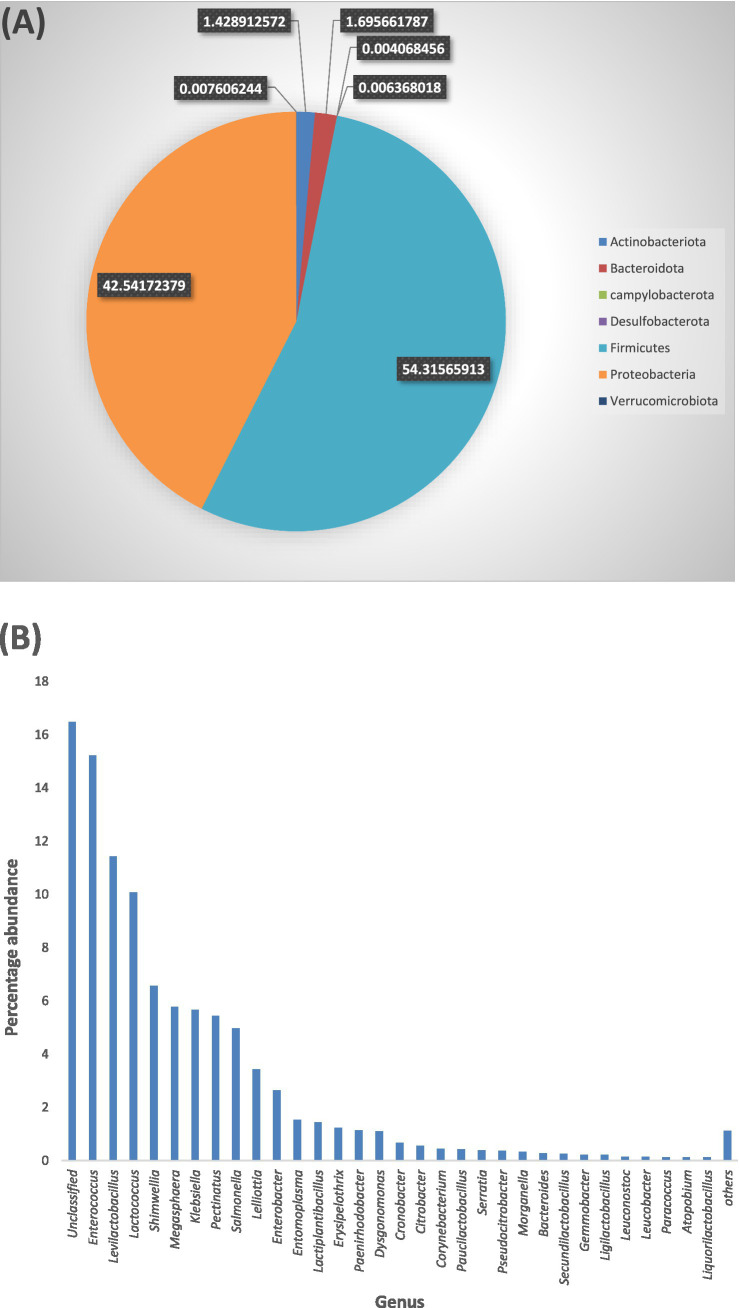
**(A)** Each section of the pie chart, having a unique colour, represents a bacterial phylum. The size of each section is indicative of the percentage abundance of the phylum. **(B)** All bacterial genera are represented in one colour (blue) on the bar chart with the length of the bars indicating the percentage abundance of each genus. To optimise the view, only genera with 0.1% abundances are shown individually on the bar chart, genera with <0.1% abundance are combined into “Others”. “Unclassified” represents the abundance of ASV counts that were not successfully resolved to genus level.

All taxa identified (100%) belonged to the kingdom Bacteria. In all, 165 genera spanning 7 phyla (*Firmicutes*, *Proteobacteria*, *Actinobacteriota*, *Bacteroidota*, *Campylobacteria*, *Desulfobacterota*, and *Verrucomicrobiota*) were identified. The dominant phyla with individual genera having a sequence abundance of 10 counts and above were *Firmicutes* (54.3% of total ASV abundance), *Proteobacteria* (42.5%), *Bacteroidota* (1.7%), and *Actinobacteriota* (1.4%) (Figure 3A). *Enterococcus*, *Levilactobacillus*, *Lactococcus*, *Shimwellia*, *Megasphaera*, *Klebsiella*, *Pectinatus*, *Salmonella*, *Lelliotia*, and *Enterobacter* were the most dominant genera, listed in decreasing order of abundance (Figure 3B), but approximately 16.4% of the ASVs were not resolved to the genus level. The ASV table (>10 average counts) generated can be seen in Supplementary Data 3.0.

#### 3.4.2 Genus-level bacterial diversity and percentage abundances in different gut segments of APW larva

##### 3.4.2.1 Taxonomic profile per gut segment

**Figure 4:**
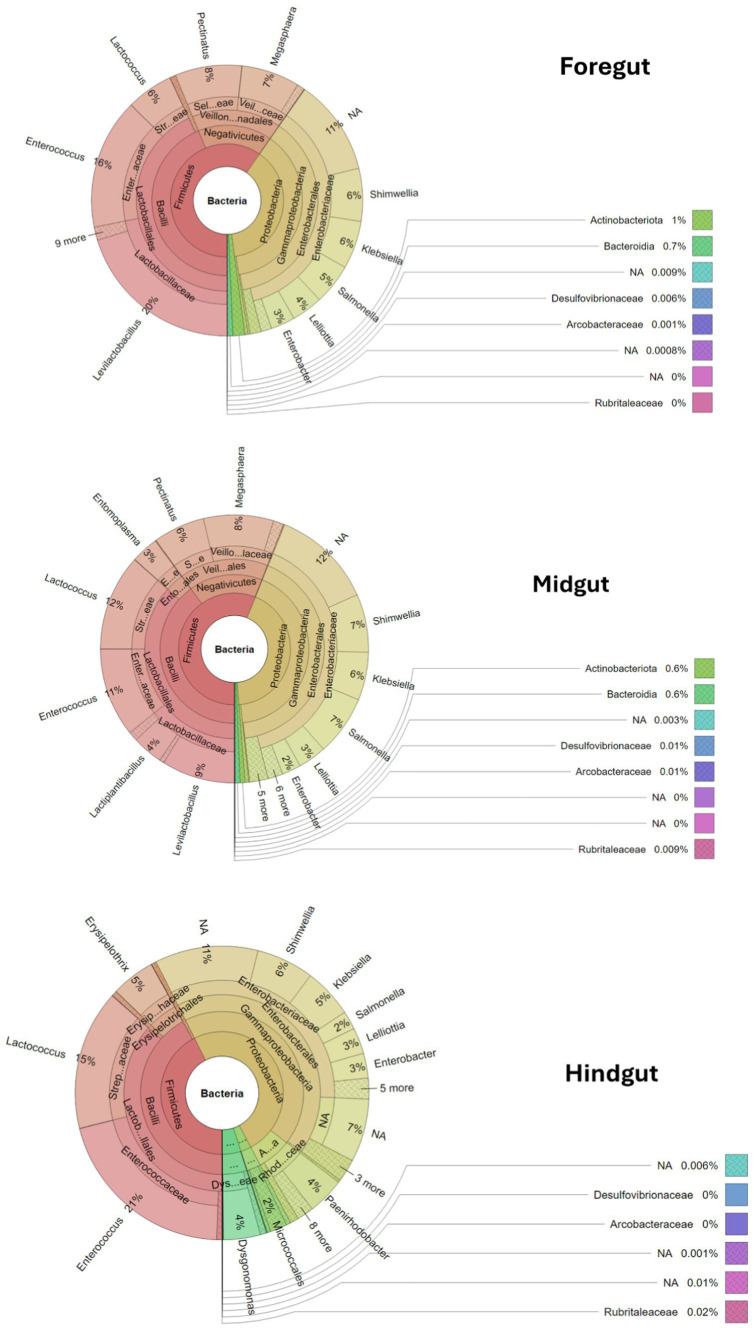
Krona plots showing the taxonomic classification of bacteria within the gut segments. Each circle represents a taxonomic level growing outwards from “Kingdom” to “genus”. Each phylum is represented by a specific colour and the different taxa at different levels within the phylum are represented by varying shades of the colour assigned to the phylum. The different genera and their percentage abundances identified can be seen in the outermost layer of the circles.

**Figure 5:**
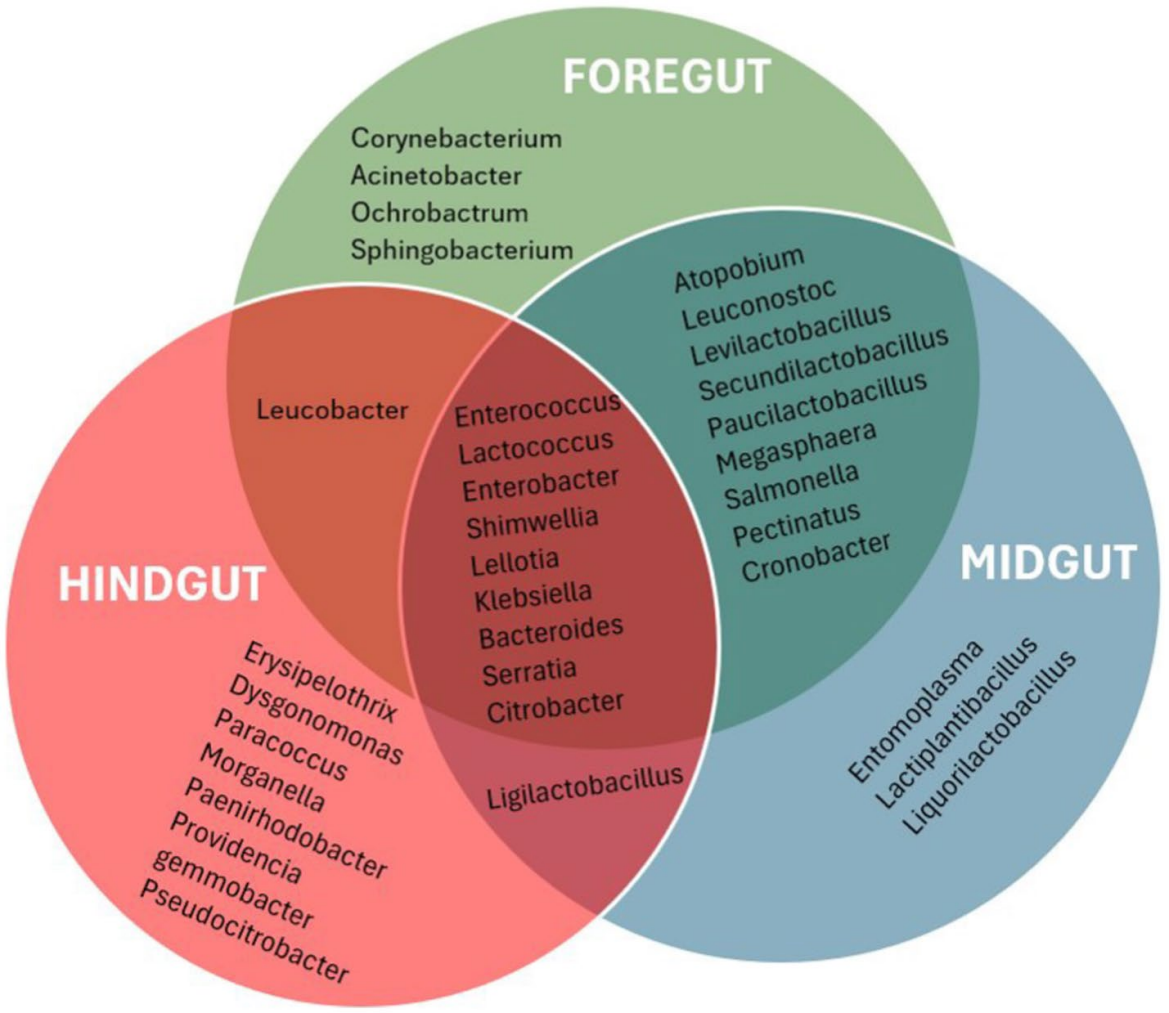
Venn diagram that presenting a visualization of the bacterial taxa found uniquely in different gut segments and those shared between the different gut segments. Only the most abundant genera with abundance ≥0.1% were used to produce the diagram for a more meaningful analysis.

The taxonomic profile and classification of bacteria identified in the foregut, midgut, and hindgut segments of the APW gut are shown in Figure 4. In each gut segment, a large percentage of the bacteria (foregut; 12%, midgut; 14%, and hindgut; 19%) were not resolved to specific bacterial genera. *Enterococcus*, *Lactococcus*, *Shimwellia*, *Lelliotia*, *Klebsiella*, *Enterobacter*, *Bacteroides*, *Serratia*, *Salmonella*, and *Citrobacter* were the genera found across all gut segments that were deemed to represent the core bacterial microbiota. The foregut and midgut shared lots of similar genera that were completely absent in the hindgut (*Megasphaera*, *Pectinatus*, *Levilactobacillus*, *Paucilactobacillus*, *Secundilactobacillus*, *Leuconostoc*, *Salmonella*, *Pectinatus*, *Cronobacter*, *and Atopobium*). The hindgut appeared to be unique, containing *Erysipelothrix*, *Morganella*, *Gemmobacter*, *Paracoccus*, *Providencia*, *Leminorella*, *Yokenella*, and *Rhizobium* exclusively, having only *Leucobacter* in common with the foregut and *Ligilactobacillus* with the midgut (Figure 5).

##### 3.4.2.2 Comparative analysis of bacterial composition across the APW gut segments

**Figure 6:**
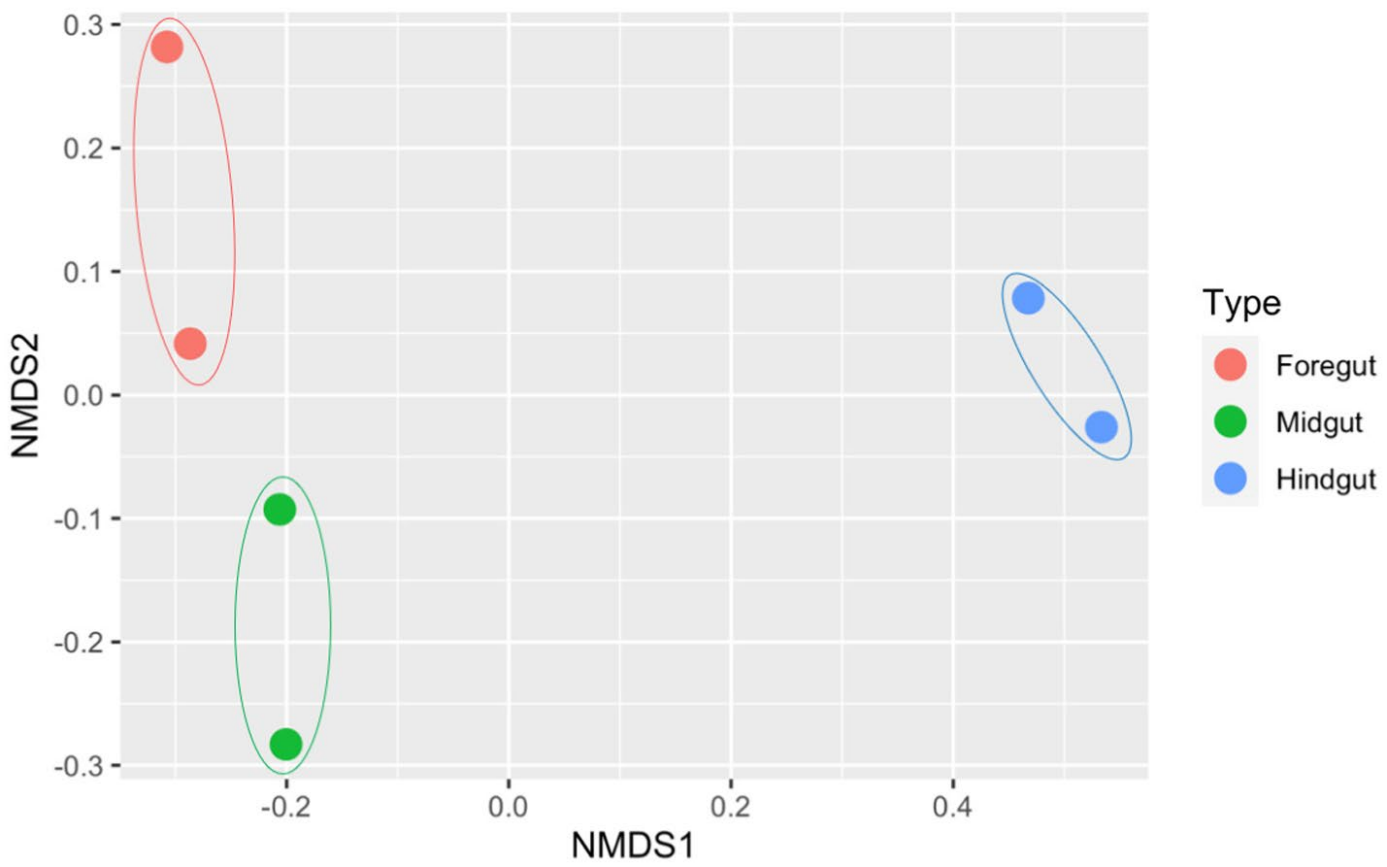
Each dot on the graph represents a particular sample and each gut segment is represented by a different colour and enclosed in a sphere to denote a specific community.

The dissimilarity between identified bacterial communities from each gut segment was calculated using the Bray–Curtis dissimilarity method, which is based on phylotype abundances and is shown in multidimensional space on a non-metric multidimensional scaling (NMDS) plot (Figure 6). Although the hindgut samples were more clearly separated from those of midgut and foregut and the points representing the same gut segment were closer to each other and separated from those representing other gut segments, there were no significant differences (PERMANOVA: *p* = 0.06667). This result might indicate that the midgut and foregut microbial communities are more similar. In contrast, the hindgut community is distinctively different, but due to the low variability amongst gut segments, there were no huge differences amongst them.

##### 3.4.2.3 Evaluation of the microbial diversity within each APW gut segment

**Figure 7:**
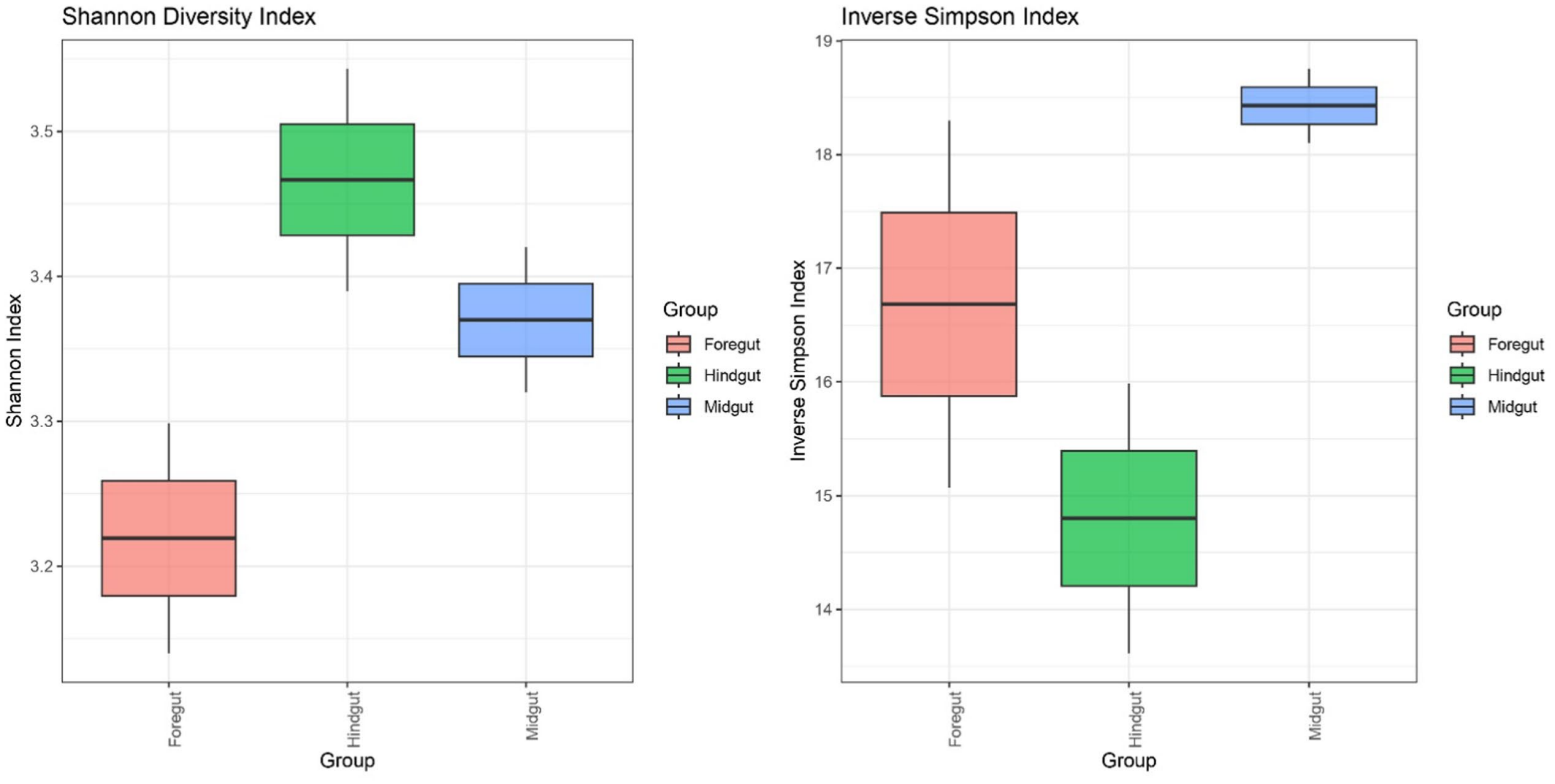
The boxes denote interquartile ranges (IQR) between the first and third quartiles (Q1-Q3) and the horizontal line inside the boxes defines the median. The whiskers which extend from Q1 and Q3 represent the lowest and highest points within 1.5-fold IQR respectively.

The species diversity was estimated using both the Shannon and inverse Simpson diversity indices (Figure 7). Whilst the Shannon index considers the richness component and rare cover of species present in the different gut segments, the inverse Simpson index emphasises the evenness component and, thus, the dominant cover species. Consequently, the Hindgut had the highest Shannon diversity index and the lowest inverse Simpson index. However, the foregut showed the lowest Shannon index, but the midgut showed the highest inverse Simpson index. Despite all these observations, there were no significant differences in the microbial diversity amongst the segments (Kruskal–Wallis test: *p* = 0.2765).

### 3.5 Diversity and relative abundance of all identified lignin-degrading bacteria in the different gut segments of APW larvae

**Figure 8:**
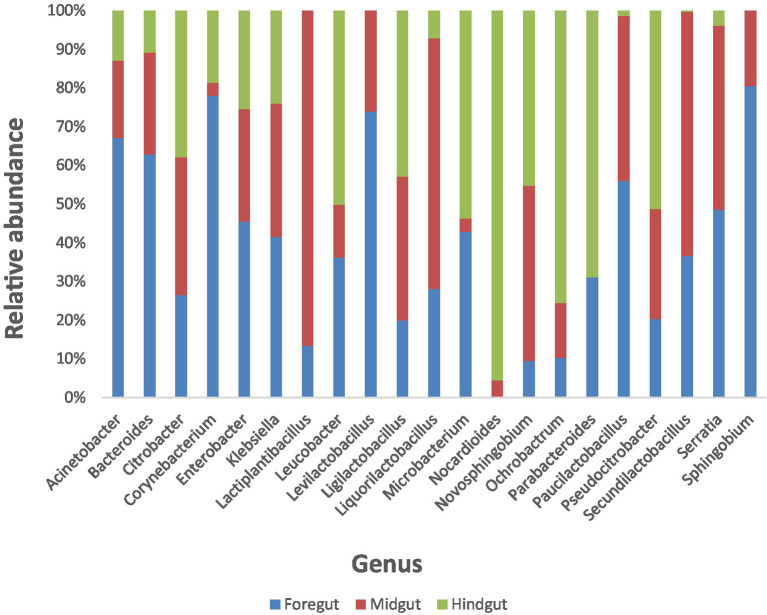
Stacked columns showing plots of relative abundances of individual lignin degrading bacteria identified in the Foregut, Midgut and Hindgut of the APW larvae as a percentage of the total gut bacterial abundance per gut segment. Each column represents a specific bacterial genus and is made up of colour coded regions (one colour for each of the gut segments), the size of which is proportional to the relative abundance of that genus in the different gut segments.

A total of 21 bacterial genera reported to have lignin-degrading ability from several literature (Table 1) were identified across the different gut segments of the African palm weevil microbiome. They represented a total of 21.49% of all identified genera within the gut. *Firmicutes* constituted 56.79 and 39.56% of the lignin degraders were from the *Proteobacteria* phylum, whilst only 2.5 and 1.13% were from the *Actinobacteriota* and *Bacteroidota* phylum, respectively. *Levilactobacillus* (46.4%), *Klebsiella* (22.9%), *Enterobacter* (10.7%), *Lactiplantibacillus* (5.9%), *Citrobacter* (2.2%), *Corynebacterium* (1.8%), *Paucilactobacillus* (1.8%), *Serratia* (1.5%), *Bacteroides* (1.1%), and *Leucobacter* (1.0%) were the most dominant lignin-degrading genera in the gut cumulatively in the listed order (Figure 8).

The foregut had 20 out of the 21 identified lignin-degrading genera (except *Norcardiodes*), adding up to a total abundance of 55.5%. The midgut followed closely, also having 20 of the identified genera (except *Parabacteroides*) and a total abundance of 32.9%. The hindgut had the least number of identified bacterial taxa (only 18 genera, with *Lactiplantibacillus*, *Levilactobacillus*, and *Sphingobium* absent) and the least total abundance of 11.5%. To facilitate comparison, we calculated the percentage abundance of each bacterial genera in each gut segment as a fraction of the total abundance of lignin-degrading taxa identified. The three gut segments shared 18 similar taxa in varying abundances, with the foregut having the highest abundance of each taxon in most cases. Overall, the foregut had the most diverse and abundant lignin-degrading genera, followed by the midgut, and the hindgut had the least.

## 4 Discussion

The quest to find enzymes capable of biological degradation of lignin as an alternative to chemical and physical methods of lignocellulose breakdown has resulted in research efforts geared towards bioprospecting these enzymes from environments where lignin degradation is known to occur naturally, such as in the guts of wood-feeding insects (Fisher and Fong, 2014; Ali et al., 2019). Recently, research on mining the microbiota of insects for genes that code for enzymes and bioactive compounds has greatly increased and is mainly being carried out via culture-independent methods (Steele et al., 2009; Hammer et al., 2015; Harnpicharnchai et al., 2007; Quince et al., 2017).

The DNA extracted from pooled gut tissues of preserved larvae had low concentration, and due to the COVID-19 lockdown, which could not allow us to return to the field (Nigeria) to collect more samples, we could only prepare duplicate samples from each gut segment pool. This posed a challenge to the attainment of at least 3 technical replicates to facilitate statistical analysis. However, being sequencing data that were subjected to quality control and filtering, the impact of using only two technical replicates is minimised, especially as the sequencing data statistics were of good quality (Table 2).

It is not just enough to have good quality data; it is also critical to assess that the sequencing did not introduce bias that will misrepresent the true composition of the microbial community after analysis, hence the need for controls and standards (Lazarevic et al., 2016; Reich et al., 2018). The negative control sample had only bacteria, which were also present in the true samples and are associated with insect guts (Egert et al., 2003; Janusz et al., 2017; Ceballos et al., 2017). This observation implies that there was no external or unexpected contamination by any foreign or exogenous bacteria. It is recommended that microbial taxa found in the control sample that correspond to genuine or biologically expected microbiota of interest should not be removed from valid samples except where they occur in higher relative abundances compared to the samples (Reich et al., 2018; Lazarevic et al., 2016).

Analysis of the mock microbial community DNA standard also found all the bacterial strains as expected, with only a few additional strains in meagre amounts of approximately 0.4% (Figure 2). Although the expected percentage abundances for the bacterial components in the community standard were slightly overrepresented as with *Bacillus*, *Staphylococcus*, *Lactobacillus*, *Escherichia*, and *Pseudomonas* or underrepresented as with *Salmonella*, *Enterococcus*, and *Listeria*. Overall, the discrepancies are minimal and validate our sequencing and bioinformatic analysis pipeline. The identified discrepancies could be because of primer and hypervariable region choice, PCR conditions, library preparation, sequencing, and data pre-processing, as well as several other variables known to introduce bias in 16S rRNA sequencing. The impact of variables cannot be eliminated but can only be minimised (Lluch et al., 2015; Jovel et al., 2016).

Microbiome studies of host-associated gut communities have identified four bacterial phyla (*Proteobacteria*, *Actinobacteria*, *Firmicutes*, and *Bacteroidota*) that predominantly colonise the guts of insects and most animals (Le, 2021; Batista-García et al., 2016; Colman et al., 2012; Engel and Moran, 2013; Franzini et al., 2016; Huang et al., 2012; Fisher and Fong, 2014). Several factors such as diet and nutrition, host taxonomy, developmental stage and habitat, seasons, gut morphology, and physicochemical conditions have been shown to affect the structure of the microbiota in most insect guts, and these findings have reported host phylogeny as being the most influential factor, with diet contributing significantly, especially in lignocellulose-feeding insects (Colman et al., 2012; Yun et al., 2014; Chew et al., 2018; Franzini et al., 2016; Huang et al., 2012; Jia et al., 2013; Tsegaye et al., 2019). Our results agree with preliminary findings of insect gut-associated bacterial communities, with the detection of the four mentioned phyla being predominant in the APW gut (Figure 3A) and similarity in taxa compared to other *Rhynchophorus* species (Valzano et al., 2012; Jia et al., 2013; Tagliavia et al., 2014; Kassim et al., 2016; Muhammad et al., 2017; Liao et al., 2020).

A large-scale cross-taxa analysis of insect-associated bacterial diversity and communities based on 137 insect specimens representing 39 species using 16S rRNA sequencing reported that, on average, most insect bacterial communities were not diverse, containing less than 8 phylotypes, and were dominated mainly by a single phylotype belonging to the phyla *Proteobacteria* or *Firmicutes* (Jones et al., 2013). However, they excluded phylotypes with less than 1% of the bacterial community in each sample, which must have eliminated many taxa with low abundance, thereby presenting a community with low diversity. Another large-scale deep sequencing effort, based on 305 individual insects belonging to 218 species, reported that the gut of insects harbours a diverse collection of bacteria (Yun et al., 2014). We identified a total of 165 bacterial genera out of which only 78, which had an abundance of 10 or more counts, were used for further analyses and gut microbiota description (Figure 3B). The situation explained above calls for caution when comparing findings across different studies, as subtle variations in methods and analysis parameters (e.g., the threshold for filtering low abundance data) if not carefully considered may lead to wrong conclusions (Knight et al., 2018; Thomas et al., 2012; Quince et al., 2017).

The most dominant genera were mostly aerobes and facultative anaerobes from the *Firmicutes* (*Enterococcus*, *Levilactobacillus*, *Lactococcus*, *Megasphaera*, and *Pectinatus*) and *Proteobacteria* phyla (*Shimwellia*, *Klebsiella*, *Salmonella*, *Lelliotia*, and *Enterobacter*). Investigations to detect the effect of different developmental stages on the gut microbiota of the red palm weevil (*Rhynchophorus ferrugineus*), a sister species to the APW (*R. phoenicis*), using non-culture-dependent 16S rRNA amplicon sequencing of the V4 hypervariable region also detected similar bacterial taxa, including *Enterobacter*, *Citrobacter*, *Serratia*, *Klebsiella*, *Lactococcus*, *Entomoplasma*, and *Erysipelothrix*, though in varying abundances (Muhammad et al., 2017). Similarly, Liao et al. (2020) identified *Enterobacter*, *Lactococcus*, and *Erysepelothrix* as dominant genera in the midgut of *R. ferrugineus*. These observations might indicate that the bacterial community has a strong phylogenetic signal, i.e., bacterial community structures are more similar amongst closely related insect species than in less related ones (Jones et al., 2013). The red palm weevil gut has also been reported to have a stable gut microbiota across all developmental stages, with differences owing more to nutrition than host taxonomy (Muhammad et al., 2017). The detection of similar abundant taxa or what could be called a “core microbiome” from our results studying the larval stage and those of studies in larval, pupal, and adult stages of *Rhynchophorus* species are in tandem with this report. There have been several other studies into the microbiota of the red palm weevil, being the most investigated species of the *Rhynchophorus* weevils, but the sequencing methods, experimental design, parameters used, and focus of these studies may not allow for an accurate comparison of total gut bacterial profile with our results (Le, 2021; Tagliavia et al., 2014; Montagna et al., 2015; Jia et al., 2013; Angzzas et al., 2016). To the best of our knowledge, this is the first attempt at profiling the microbiome of *Rhynchophorus phoenicis*, and there is no published record of gut microbiota studies of other *Rhynchophorus* relatives such as *R. cruentatus*, *R. palmarum*, and *R. vulneratus*.

All the gut segments of the APW larvae shared an appreciable number of core taxa, whilst the foregut and midgut particularly had more taxa in common, hence exhibiting greater similarity in community structure compared to the hindgut, which was more compositionally unique (Figures 4, 5). Alpha diversity estimation of the different gut segments using the Shannon diversity and inverse Simpson indices visualised by box plots (Figure 6) shows that the hindgut harboured more diverse bacterial taxa, followed by the midgut and then the foregut (the higher the Shannon index, the higher the diversity), corroborating the findings presented in Figure 5. However, taxa distribution within the foregut was more even than in the hindgut, with the most uneven distribution found in the midgut (the wider the size of the box, i.e., the interquartile range, the less even the distribution) (Liu et al., 2017; Li et al., 2018). Although the statistics do not present any significant differences, probably due to having only two technical replicates, the visual representation from Figure 5 and the alpha diversity analyses above support this assertion. These observations support the submission made by other researchers that the difference in morphology (shape, size) and physicochemical conditions (oxygen, temperature, pH, and mineral elements) within each gut compartment affects the structure of the microbial community that exists in it (Engel and Moran, 2013; Valzano et al., 2012; Egert et al., 2003; Chew et al., 2018).

In the absence of an existing database of lignin-degrading bacteria to our knowledge, we compiled information from many pieces of literature stemming from research where bacteria have been implicated or tentatively confirmed to be associated with the decomposition of any part of the lignin molecule (Table 1). We used that list as a reference document, and any bacteria on that list that is identified within the gut of the APW was considered as lignin-degrading bacteria. The lignin degraders constituted 29.5% of the total taxa identified within the larval gut and were drawn from the phyla *Firmicutes* predominantly (56.8%), *Proteobacteria* (39.5%), *Actinobacteriota* (2.5%), and *Bacteroidota* (1.1%). The dominance of lignin-degrading bacteria belonging to the *Proteobacteria* and *Firmicutes* phyla has been consistently reported in all previous research we have accessed on best-characterised lignin-degrading bacteria along with other phyla such as *Bacteriodota* and *Actinobacteria* (see a detailed list of sources in Table 1).

The distribution of the 21 lignin degradation-associated bacterial genera (*Klebsiella*, *Enterobacter*, *Citrobacter*, *Corynebacterium*, *Serratia*, *Bacteroides*, *Leucobacter*, *Acinetobacter*, *Ochrobactrum*, *Microbacterium*, *Sphingobium*, *Novosphingobium*, *Thermomonas*, *Sphingomonas*, *Delftia*, and *Pseudomonas*) across the different gut segments and their relative abundance per segment is presented in Figure 8.

The physical and chemical characteristics of the major components of lignocellulose and the physicochemical conditions such as pH and oxygen availability within the insect’s gut are the major determining factors responsible for the distribution pattern of the lignocellulose-degrading machinery in insects (Sun and Zhou, 2011; Yun et al., 2014). In a detailed morphological and histological description of the APW digestive tract, it was reported to have a foregut, midgut, and hindgut. The foregut is the largest gut segment made up of the buccal cavity, oesophagus, crop, and proventriculus, which are all adapted for intake, mechanical grinding, storage, and onward passage of food to the midgut. The midgut and hindgut are structurally and functionally adapted for the digestion of food, assimilation of nutrients, and excretion of wastes (Omotoso, 2013). The same alimentary tract structure was reported for *R. ferrugineus* by Harris et al. (2015) in a similar study of the morphology and histology of the RPW larval gut.

Different segments of the gut have unique characteristics, which make them susceptible to colonisation by different types of bacteria (Engel and Moran, 2013). The microbiome within a gut compartment is affected by morphology, which varies as insects metamorphose from one developmental stage to the next in most insect orders. The size and shape of the gut additionally influence the availability of oxygen due to the partial pressure of oxygen from the external environment, which in turn determines the metabolism of the inhabitant bacteria (Yun et al., 2014). For effective utilisation of lignocellulose by wood-feeding insects for energy, depolymerization of lignin must occur first to grant access to hydrolytic enzymes to release the stored-up energy in the carbohydrate polymers, cellulose and hemicellulose (Sun and Zhou, 2011; Kumar et al., 2017a; Silva et al., 2018). Lignin degradation is an aerobic oxidation process requiring oxidative enzymes such as peroxidases, oxidases, and laccases; hence, it is believed that these reactions are most likely to occur in the foregut, being the anterior part of the gut closest to the external environment where oxygen supply is highest (Chew et al., 2018; Sun and Zhou, 2011). In contrast, the midgut and hindgut have been reported as the sites for cellulose and hemicellulose degradation (Egert et al., 2003; Sun and Zhou, 2011; Chew et al., 2018; Yun et al., 2014). These fermentative processes occur by anaerobic hydrolysis; thus, it is reasonable to expect the degradation machinery to be domiciled in the interior, anaerobic compartments of the gut that are farther away from oxygen supply.

The presence almost exclusively of aerobic and facultatively anaerobic bacteria (except *Bacteroides* alone, which is anaerobic) within the gut of APW and the specific abundance distribution of lignin-degrading bacteria within each gut compartment demonstrates the adaptability of the APW larvae to digesting its diet and suggests where lignin degradation most likely occurs (Khiyami and Alyamani, 2008). Olsson (2016) has reported that the gut of mammals houses more obligate and facultative anaerobes, whilst insect guts have a prevalence of aerobes and facultative anaerobes and a large variety of lignin-associated enzymes.

From our results, the foregut of APW larvae possessed the most diverse and highest percentage abundance of lignin-degrading phylotypes compared to the midgut and hindgut. The presence of the proventriculus as part of the foregut of the APW’s digestive tract shows their adaptation to their food source (lignocellulosic palm tissues) and explains their ability to offer some sort of mechanical pre-treatment to the lignin in their diet as a first step towards extracting energy from the polysaccharides that occur in the later parts of the gut following a logical order. A similar investigation of bacterial community structure in the foregut, midgut, and hindgut of the wood-feeding termite *bulbitermes* sp. by Chew et al. (2018) suggested that lignin degradation was most probably held in the foregut due to the significantly higher relative abundance of the lignin-degrading bacteria, *Actinomycetales*, in the foregut compared to the other segments. They further justified their assertion following predictive functional profiling where they found energy and co-factor metabolism predominantly occurring in the hindgut, whereas oxidative xenobiotic degradation reactions (which are related to lignin degradation reactions) occurred mostly in the foregut. Overall, our results, supported by the studies of Chew et al. (2018), and the several other pieces of literature cited above, seem to rationalise the foregut of the APW larvae as being the site for lignin degradation prior to cellulose and hemicellulose degradation in the other gut compartments.

## 5 Conclusion

Our study, which represents the first known metaprofiling effort of the bacteria colonising the gut of the African palm weevil, *R. phoenicis*, to date, has revealed great similarity in bacterial community structure with those identified in most insects, specifically with the bacterial microbiota of the phylogenetically related red palm weevil, *Rhynchophorus ferrugineus*. An appreciable number of lignin-degrading bacteria within the larval gut suggests an immense potential for the discovery of lignin-degrading genes and enzymes. Furthermore, lignin degradation in the African palm weevil is believed to be domiciled in its foregut due to the presence of a proventriculus that serves to mechanically decrease the structural complexity of lignocellulose as a first step towards degradation and the greatest abundance of mostly aerobic and facultatively anaerobic bacteria capable of oxidatively decomposing lignin predominating the foregut. Our findings point towards the gut of the African palm weevil being a reservoir that harbours a consortium of bacteria capable of lignin degradation/modification from which lignin-degrading genes and enzymes can be harvested.

## Data Availability

The datasets presented in this study can be found in online repositories. The names of the repository/repositories and accession number(s) can be found at: https://www.ebi.ac.uk/biostudies/arrayexpress/studies/E-MTAB-13719?key=639be3f8-41bb-40e1-970c-44cc13632281, E-MTAB-13719.
