# Supplementary material for: Bacterial community profiling and identification of bacteria with lignin-degrading potential in different gut segments of African palm weevil larvae (Rhynchophorus phoenicis)
Source: Front Microbiol. 2025 Jan 3;15:1401965. doi: 10.3389/fmicb.2024.1401965 (PMC11739302; doi:10.3389/fmicb.2024.1401965)
Supplement: Supplementary file 1 [file Supplementary_file_1.pdf]

Supplementary Material

Bacterial community profiling and identification of bacteria with lignin degrading potential in different gut segments of the African palm weevil larvae (*Rhynchophorus phoenicis*)

Jessica Lenka [*], Enrique González-Tortuero, Shweta kuba, Natalie Ferry*

*** Correspondence: n.ferry@salford.ac.uk**

Supplementary Data

1.0 NAP buffer recipe (1 Litre)

Weigh and dissolve the following reagents in 800mls distilled water in a volumetric flask.

**Reagent**	**Amount required.**
EDTA	7.44g
Sodium citrate trisodium salt dihydrate	7.35g
Ammonium sulfate	700g

Use H_2SO_4 to adjust the pH of the solution to 5.2 and make up to 1L with distilled water.

2.0 Field collection of APW larvae

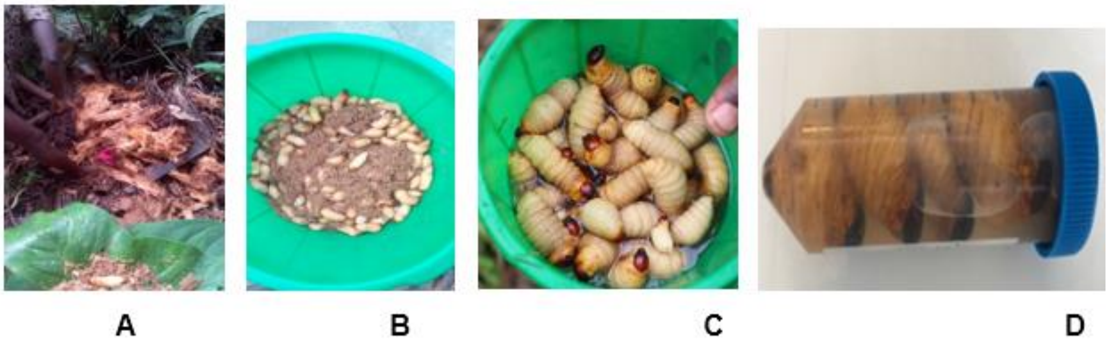

Figure 2: Pictures from larvae collection and preservation
A: Felling and hacking down of palm tree to access the APW larvae; B: Harvested larvae in a plastic bowl with chopped parts from palm tree trunk; C: Surface sterilization of APW larvae; D: Sterilized larvae packaged in NAP buffer

3.0 ASV Table

						Table 1: Amplicon Sequence Variants (ASVs)									
	Kingdom	Phylum	Class	Order	Family	Genus	Foregut1	Foregut2	Foregut AV	Hindgut1	Hindgut2	Hindgut AV	Midgut1	Midgut2	Midgut AV
ASV1	Bacteria	Firmicut	Bacilli	Lactob	Enteroc	Enterococcus	18712	15207	16959.5	11854	10517	11185.5	10079	6836	8457.5
ASV2	Bacteria	Firmicut	Bacilli	Lactob	Streptoc	Lactococcus	7809	5353	6581	11871	9411	10641	13303	8712	11007.5
ASV3	Bacteria	Proteoba	Gamm	Entero	Enteroba	Shimwellia	6872	7978	7425	4931	3942	4436.5	7189	6153	6671
ASV4	Bacteria	Firmicut	Negati	Veillor	Veillone	Megasphaera	6831	8085	7458	77	108	92.5	10535	4196	7365.5
ASV5	Bacteria	Proteoba	Gamm	Entero	Enteroba	Salmonella	5244	6223	5733.5	1279	1588	1433.5	5579	8154	6866.5
ASV6	Bacteria	Firmicut	Negati	Veillor	Selenom	Pectinatus	6983	8938	7960.5	66	56	61	7088	2917	5002.5
ASV7	Bacteria	Firmicut	Bacilli	Lactob	Lactoba	Levilactobacillus	12686	5893	9289.5	0	0	0	3780	2819	3299.5
ASV8	Bacteria	Proteoba	Gamm	Entero	Enteroba	NA	3684	6578	5131	2354	2916	2635	4633	3881	4257
ASV9	Bacteria	Proteoba	Gamm	Entero	Enteroba	NA	4908	5665	5286.5	830	1286	1058	4064	2744	3404
ASV10	Bacteria	Proteoba	Gamm	Entero	Enteroba	Lelliottia	3329	5249	4289	1721	2222	1971.5	3497	2735	3116
ASV11	Bacteria	Proteoba	Gamm	Entero	Enteroba	Klebsiella	2541	4585	3563	1268	2192	1730	3735	1760	2747.5
ASV12	Bacteria	Firmicut	Bacilli	Lactob	Lactoba	Levilactobacillus	7788	3966	5877	0	0	0	2240	1714	1977
ASV13	Bacteria	Proteoba	Gamm	Entero	NA	NA	1378	1607	1492.5	4433	5199	4816	1744	1225	1484.5
ASV14	Bacteria	Proteoba	Gamm	Entero	Enteroba	NA	1879	2450	2164.5	3666	2276	2971	2062	3020	2541
ASV15	Bacteria	Proteoba	Gamm	Entero	Enteroba	Enterobacter	2789	3564	3176.5	1963	1622	1792.5	2799	1231	2015
ASV16	Bacteria	Firmicut	Bacilli	Lactob	Lactoba	Levilactobacillus	6343	3179	4761	0	0	0	1861	1513	1687
ASV18	Bacteria	Firmicut	Bacilli	Lactob	Lactoba	Levilactobacillus	5279	2600	3939.5	12	0	6	1708	1256	1482
ASV20	Bacteria	Firmicut	Bacilli	Entom	Entomo	Entomoplasma	119	1208	663.5	106	680	393	556	5187	2871.5
ASV23	Bacteria	Proteoba	Gamm	Entero	Enteroba	Klebsiella	1074	1968	1521	334	810	572	1940	791	1365.5
ASV24	Bacteria	Firmicut	Bacilli	Lactob	Enteroc	Enterococcus	1234	1416	1325	117	66	91.5	1527	765	1146
ASV25	Bacteria	Firmicut	Bacilli	Lactob	Lactoba	Lactiplantibacillus	525	331	428	0	12	6	3477	2122	2799.5
ASV26	Bacteria	Proteoba	Gamm	Entero	Enteroba	Klebsiella	845	1318	1081.5	702	1038	870	1168	511	839.5
ASV31	Bacteria	Firmicut	Negati	Veillor	Veillone	NA	969	1227	1098	14	18	16	1841	574	1207.5
ASV32	Bacteria	Firmicut	Negati	Veillor	Selenom	Pectinatus	1212	1580	1396	0	0	0	1291	485	888
ASV33	Bacteria	Proteoba	Alphap	Rhodo	Rhodoba	Paenirhodobacter	135	111	123	1742	1794	1768	227	59	143
ASV34	Bacteria	Actinoba	Actinol	Coryne	Coryneb	Corynebacterium	1037	945	991	410	65	237.5	58	27	42.5
ASV36	Bacteria	Firmicut	Negati	Veillor	Veillone	Megasphaera	559	713	636	0	0	0	802	327	564.5
ASV37	Bacteria	Firmicut	Bacilli	Lactob	Lactoba	Paucilactobacillus	764	617	690.5	0	19	9.5	623	356	489.5
ASV38	Bacteria	Bacteroi	Bacter	Bacter	Dysgonc	Dysgonomonas	5	18	11.5	829	1144	986.5	27	308	167.5
ASV39	Bacteria	Proteoba	Gamm	Entero	Enteroba	NA	357	400	378.5	195	84	139.5	845	334	589.5
ASV40	Bacteria	Proteoba	Gamm	Entero	Enteroba	Cronobacter	280	566	423	217	136	176.5	516	423	469.5
ASV41	Bacteria	Proteoba	Gamm	Entero	Enteroba	Klebsiella	279	498	388.5	180	354	267	533	257	395
ASV42	Bacteria	Proteoba	Gamm	Entero	Enteroba	Cronobacter	292	475	383.5	124	51	87.5	453	309	381
ASV43	Bacteria	Proteoba	Gamm	Entero	Enteroba	NA	335	397	366	249	222	235.5	383	216	299.5
ASV44	Bacteria	Firmicut	Bacilli	Lactob	Lactoba	Lactiplantibacillus	156	78	117	0	0	0	934	556	745
ASV45	Bacteria	Proteoba	Gamm	Entero	Yersinia	Serratia	0	40	20	437	310	373.5	569	196	382.5
ASV46	Bacteria	Proteoba	Alphap	Rhodo	Rhodoba	Paenirhodobacter	66	41	53.5	535	655	595	69	26	47.5
ASV47	Bacteria	Proteoba	Gamm	Entero	Enteroba	Pseudocitrobacter	0	239	119.5	185	512	348.5	54	376	215
ASV50	Bacteria	Firmicut	Bacilli	Lactob	Lactoba	Ligilactobacillus	65	101	83	129	535	332	229	128	178.5
ASV52	Bacteria	Firmicut	Bacilli	Lactob	Lactoba	Secundilactobacillus	185	252	218.5	4	0	2	404	216	310
ASV53	Bacteria	Actinoba	Actinol	Bifidob	Bifidoba	NA	182	195	188.5	65	82	73.5	297	147	222
ASV54	Bacteria	Actinoba	Actinol	Microc	Microba	Leucobacter	137	144	140.5	246	125	185.5	62	44	53
ASV56	Bacteria	Proteoba	Gamm	Entero	Morgane	Morganella	29	36	32.5	364	326	345	46	33	39.5
ASV57	Bacteria	Proteoba	Gamm	Entero	Enteroba	Citrobacter	33	57	45	276	281	278.5	117	59	88
ASV58	Bacteria	Firmicut	Bacilli	Entom	Entomo	Entomoplasma	0	157	78.5	0	64	32	0	590	295
ASV59	Bacteria	Firmicut	Bacilli	Lactob	Lactoba	Leuconostoc	233	122	177.5	31	26	28.5	189	167	178
ASV60	Bacteria	Proteoba	Alphap	Rhizob	Rhizobia	NA	45	53	49	279	272	275.5	66	27	46.5
ASV61	Bacteria	Proteoba	Gamm	Entero	Enteroba	Enterobacter	143	196	169.5	97	73	85	164	65	114.5
ASV65	Bacteria	Proteoba	Gamm	Entero	Enteroba	NA	74	118	96	0	0	0	341	133	237
ASV66	Bacteria	Proteoba	Gamm	Entero	Enteroba	Citrobacter	0	34	17	217	250	233.5	98	40	69
ASV67	Bacteria	Proteoba	Gamm	Entero	Enteroba	NA	42	45	43.5	171	252	211.5	77	47	62
ASV70	Bacteria	Firmicut	Bacilli	Lactob	Enteroc	Enterococcus	99	73	86	101	75	88	64	51	57.5
ASV72	Bacteria	Firmicut	Bacilli	Lactob	Enteroc	Enterococcus	16	16	16	35	68	51.5	334	119	226.5
ASV74	Bacteria	Proteoba	Alphap	Rhodo	Rhodoba	Gemmobacter	41	49	45	170	254	212	28	19	23.5
ASV76	Bacteria	Proteoba	Gamm	Entero	Enteroba	Lelliottia	0	203	101.5	0	96	48	59	164	111.5
ASV77	Bacteria	Firmicut	Bacilli	Lactob	Enteroc	Enterococcus	43	0	21.5	277	176	226.5	12	0	6
ASV80	Bacteria	Proteoba	Gamm	Entero	Enteroba	Citrobacter	106	178	142	0	0	0	136	55	95.5
ASV81	Bacteria	Proteoba	Alphap	Rhodo	Rhodoba	Gemmobacter	21	18	19.5	189	177	183	44	0	22
ASV82	Bacteria	Actinoba	Coriob	Coriob	Atopobia	Atopobium	128	97	112.5	3	2	2.5	134	81	107.5
ASV83	Bacteria	Bacteroi	Bacter	Sphing	Sphingol	Sphingobacterium	97	88	92.5	64	74	69	85	27	56
ASV84	Bacteria	Firmicut	Bacilli	Lactob	Lactoba	Liquorilactobacillus	55	75	65	0	0	0	162	134	148
ASV85	Bacteria	Proteoba	Gamm	Entero	Enteroba	Pseudocitrobacter	61	87	74	48	102	75	34	92	63
ASV87	Bacteria	Firmicut	Negati	Veillor	Veillone	Megasphaera	79	101	90	0	0	0	147	57	102
ASV88	Bacteria	Proteoba	Gamm	Entero	Morgane	Morganella	0	24	12	175	170	172.5	0	0	0
ASV89	Bacteria	Proteoba	Gamm	Entero	Enteroba	Citrobacter	105	88	96.5	0	0	0	129	42	85.5
ASV90	Bacteria	Bacteroi	Bacter	Flavob	Weekse	NA	169	127	148	16	0	8	37	14	25.5
ASV93	Bacteria	Actinoba	Actinol	Actino	Actinom	NA	17	13	15	153	134	143.5	15	10	12.5
ASV94	Bacteria	Proteoba	Gamm	Burkho	Comam	NA	49	34	41.5	54	147	100.5	37	11	24
ASV95	Bacteria	Proteoba	Gamm	Entero	Enteroba	Klebsiella	78	78	78	0	0	0	124	45	84.5
ASV98	Bacteria	Proteoba	Gamm	Pseud	Moraxel	Acinetobacter	111	119	115	13	23	18	36	14	25
ASV99	Bacteria	Proteoba	Gamm	Entero	Enteroba	Mangrovibacter	24	55	39.5	147	84	115.5	0	0	0
ASV104	Bacteria	Firmicut	Bacilli	Lactob	Lactoba	Secundilactobacillus	39	37	38	0	0	0	138	72	105
ASV105	Bacteria	Bacteroi	Bacter	Flavob	Weekse	Moheibacter	95	79	87	28	37	32.5	32	7	19.5
ASV107	Bacteria	Proteoba	Gamm	Entero	Enteroba	Citrobacter	23	33	28	26	51	38.5	87	37	62
ASV113	Bacteria	Proteoba	Gamm	Entero	Enteroba	Klebsiella	45	64	54.5	0	29	14.5	56	36	46
ASV114	Bacteria	Actinoba	Coriob	Coriob	Atopobia	Atopobium	49	59	54	0	0	0	86	34	60
ASV116	Bacteria	Proteoba	Gamm	Entero	Enteroba	Citrobacter	49	30	39.5	0	0	0	93	52	72.5
ASV121	Bacteria	Proteoba	Alphap	Sphing	Sphingo	Sphingobium	11	13	12	20	19	19.5	16	0	8
ASV123	Bacteria	Proteoba	Gamm	Entero	Enteroba	NA	55	50	52.5	0	0	0	65	29	47
ASV125	Bacteria	Proteoba	Gamm	Entero	Enteroba	Citrobacter	0	52	26	40	45	42.5	33	25	29
ASV127	Bacteria	Firmicut	Bacilli	Staphy	Staphylc	NA	146	46	96	0	0	0	0	0	0
ASV130	Bacteria	Proteoba	Alphap	Rhodo	Rhodoba	Paracoccus	11	30	20.5	74	71	72.5	0	0	0
ASV131	Bacteria	Bacteroi	Bacter	Bacter	Bacteroi	Bacteroides	0	20	10	0	25	12.5	75	64	69.5
ASV132	Bacteria	Bacteroi	Bacter	Flavob	Weekse	NA	71	67	69	0	15	7.5	29	0	14.5
ASV135	Bacteria	Proteoba	Gamm	Entero	Enteroba	Klebsiella	30	58	44	28	56	42	0	0	0
ASV138	Bacteria	Proteoba	Gamm	Pseud	Moraxel	Acinetobacter	47	43	45	6	8	7	32	17	24.5
ASV140	Bacteria	Bacteroi	Bacter	Bacter	Bacteroi	Bacteroides	39	0	19.5	49	25	37	38	0	19
ASV141	Bacteria	Proteoba	Alphap	Rhizob	Rhizobia	Ochrobactrum	11	10	10.5	48	51	49.5	24	5	14.5
ASV142	Bacteria	Actinoba	Actinol	Coryne	Nocardi	Gordonia	33	30	31.5	42	19	30.5	15	9	12
ASV143	Bacteria	Bacteroi	Bacter	Flavob	Weekse	NA	46	67	56.5	0	11	5.5	23	0	11.5
ASV155	Bacteria	Firmicut	Clostri	Oscillo	Ruminoc	Faecalibacterium	39	0	19.5	51	0	25.5	0	0	0
ASV157	Bacteria	Proteoba	Gamm	Entero	Enteroba	Pseudocitrobacter	0	26	13	17	47	32	0	20	10
ASV158	Bacteria	Firmicut	Negati	Veillor	Selenom	Pectinatus	24	25	24.5	0	0	0	44	16	30
ASV159	Bacteria	Proteoba	Gamm	Entero	Enteroba	Citrobacter	0	26	13	0	0	0	57	26	41.5
ASV171	Bacteria	Bacteroi	Bacter	Bacter	Prevotel	Prevotella_9	0	86	43	0	0	0	0	0	0
ASV173	Bacteria	Bacteroi	Bacter	Bacter	Bacteroi	Bacteroides	58	0	29	0	0	0	0	0	0
ASV174	Bacteria	Actinoba	Actinol	Bifidob	Bifidoba	Alloscardovia	22	28	25	0	0	0	34	0	17
ASV175	Bacteria	Proteoba	Gamm	Entero	Enteroba	Enterobacter	0	84	42	0	0	0	0	0	0
ASV180	Bacteria	Proteoba	Gamm	Entero	Enteroba	Yokenella	14	13	13.5	10	7	8.5	11	23	17
ASV207	Bacteria	Bacteroi	Bacter	Chitin	NA	NA	26	25	25.5	0	5	2.5	6	0	3
ASV208	Bacteria	Actinoba	Actinol	Bifidob	Bifidoba	Bifidobacterium	11	14	12.5	9	13	11	12	0	6
ASV215	Bacteria	Proteoba	Alphap	Rhizob	Rhizobia	Allorhizobium-Neorhizobium-Pararhizobium-Rh	13	18	15.5	12	11	11.5	0	0	0
ASV216	Bacteria	Proteoba	Gamm	Pseud	Moraxel	Acinetobacter	12	22	17	8	4	6	7	0	3.5
ASV218	Bacteria	Firmicut	Bacilli	Lactob	Lactoba	Liquorilactobacillus	18	16	17	0	0	0	8	10	9
ASV223	Bacteria	Bacteroi	Bacter	Bacter	Prevotel	Prevotella_9	0	47	23.5	0	0	0	0	0	0
ASV225	Bacteria	Proteoba	Gamm	Burkho	Neisseri	Snodgrassella	26	8	17	0	0	0	10	0	5
ASV226	Bacteria	Proteoba	Gamm	Entero	Enteroba	Klebsiella	22	0	11	22	0	11	0	0	0
ASV227	Bacteria	Actinoba	Actinol	Bifidob	Bifidoba	NA	13	13	13	0	0	0	18	0	9
ASV234	Bacteria	Firmicut	Clostri	Peptos	Peptostr	Romboutsia	9	19	14	0	0	0	0	14	7
ASV235	Bacteria	Bacteroi	Bacter	Bacter	Prevotel	Prevotella_9	0	41	20.5	0	0	0	0	0	0
ASV244	Bacteria	Actinoba	Actinol	Microc	Microba	Microbacterium	32	6	19	0	0	0	0	0	0
ASV247	Bacteria	Bacteroi	Bacter	Flavob	Weekse	NA	36	0	18	0	0	0	0	0	0
ASV248	Bacteria	Bacteroi	Bacter	Bacter	Prevotel	Prevotella_7	19	14	16.5	0	0	0	0	3	1.5
ASV256	Bacteria	Proteoba	Gamm	Pseud	Moraxel	Acinetobacter	10	10	10	0	0	0	12	0	6
ASV259	Bacteria	Bacteroi	Bacter	Bacter	Prevotel	Prevotella_9	0	31	15.5	0	0	0	0	0	0
ASV261	Bacteria	Proteoba	Gamm	Entero	Yersinia	Serratia	30	0	15	0	0	0	0	0	0
ASV262	Bacteria	Actinoba	Actinol	Microc	Dermab	Brachybacterium	15	10	12.5	5	0	2.5	0	0	0
ASV263	Bacteria	Actinoba	Actinol	Microc	Microba	Microbacterium	15	10	12.5	0	0	0	5	0	2.5
ASV265	Bacteria	Bacteroi	Bacter	Bacter	Bacteroi	Bacteroides	22	0	11	0	7	3.5	0	0	0
ASV272	Bacteria	Actinoba	Actinol	Microc	Microba	Pseudoclavibacter	25	0	12.5	0	0	0	0	0	0
ASV274	Bacteria	Actinoba	Actinol	Coryne	Nocardi	Gordonia	24	0	12	0	0	0	0	0	0
ASV279	Bacteria	Firmicut	Clostri	Lachn	Lachnos	Anaerostipes	0	22	11	0	0	0	0	0	0
ASV285	Bacteria	Bacteroi	Bacter	NA	NA	NA	10	11	10.5	0	0	0	0	0	0

4.0 Ethical approval

University of Salford MANCHESTER

Research, Innovation and Academic Engagement Ethical Approval Panel

Research Centres Support Team
G0.3 Joule House
University of Salford
M5 4WT

T +44(0)161 295 5278

www.salford.ac.uk/

27 June 2018

Jessica Lenka

Dear Jessica,

RE: ETHICS APPLICATION STR1718- 46 : Identification and characterisation of novel lignin degrading enzymes through metagenomics.

Based on the information you provided, I am pleased to inform you that your application STR1718-46 has been approved.

If there are any changes to the project and/ or its methodology, please inform the Panel as soon as possible by contacting S&T-ResearchEthics@salford.ac.uk

Yours sincerely,

APHigham.

Dr Anthony Higham
Chair of the Science & Technology Research Ethics Panel
